# A Survey of Smartphone-Based Indoor Positioning System Using RF-Based Wireless Technologies

**DOI:** 10.3390/s20247230

**Published:** 2020-12-17

**Authors:** Santosh Subedi, Jae-Young Pyun

**Affiliations:** Department of Information and Communication Engineering, Chosun University, Kwangju 501-759, Korea; santoshmsubedi@chosun.kr

**Keywords:** indoor positioning system, fingerprinting localization, Bluetooth low energy, Wi-Fi, performance metrics, positioning algorithms

## Abstract

In recent times, social and commercial interests in location-based services (LBS) are significantly increasing due to the rise in smart devices and technologies. The global navigation satellite systems (GNSS) have long been employed for LBS to navigate and determine accurate and reliable location information in outdoor environments. However, the GNSS signals are too weak to penetrate buildings and unable to provide reliable indoor LBS. Hence, GNSS’s incompetence in the indoor environment invites extensive research and development of an indoor positioning system (IPS). Various technologies and techniques have been studied for IPS development. This paper provides an overview of the available smartphone-based indoor localization solutions that rely on radio frequency technologies. As fingerprinting localization is mostly accepted for IPS development owing to its good localization accuracy, we discuss fingerprinting localization in detail. In particular, our analysis is more focused on practical IPS that are realized using a smartphone and Wi-Fi/Bluetooth Low Energy (BLE) as a signal source. Furthermore, we elaborate on the challenges of practical IPS, the available solutions and comprehensive performance comparison, and present some future trends in IPS development.

## 1. Introduction

Localization is the discovery of a location of a user, which is a basic need for pervasive applications such as behavior recognition, smart medication, and smart building that require accurate position information of the users to yield accurate and timely services. The demand for location-based service (LBS) has gradually increased at present owing to the rapid development and popularization of smart devices and technologies. The extensively used technology for LBS is global navigation satellite systems (GNSS). GNSS based LBS are employed in consumer products such as vehicle navigation, navigation services on the smartphone and geotagging, and scientific observation systems such as variations in the earth’s rotation and monitoring the tectonic plates. The global positioning system (GPS) [1], the Russian GLONASS [2], the European GALILEO [3], and the Chinese BeiDou Satellite Navigation System [4] are some emerging GNSS. Although GNSS-based LBS are widely used, their performance is limited to the outdoor environment only. Besides, indoor environments are often complex due to obstacles and environment changes, resulting in signal fluctuation or noise. Hence, it invites extensive research on indoor LBS or indoor positioning system (IPS) with alternative wireless technology.

The IPS is realized with the different signal sources or access points (APs). There are two choices of signal source: Either already deployed APs like Wi-Fi [5,6] and geomagnetic fields [7] or deploy a new signal source like Bluetooth low energy (BLE) and ultra-wideband (UWB) radio signal tags. The signal-free solutions in IPS is a dead-reckoning technique that uses off-the-shelf mobile sensors to detect position changes. Some of the wireless signal measuring principles in IPS are received signal strength (RSS), time of arrival (TOA), time difference of arrival (TDOA), and angle of arrival (AOA). RSS has been widely used for designing IPS owing to its nonrequirement of extra hardware and easy implementation. Indoor LBS is applicable at asset management, people tracking, trade fairs and events, etc.

As the indoor environment is complex, multipath propagation and shadowing effect on a radio signal is common [8]. Hence, the received signal can contain line-of-sight (LOS) and non-line-of-sight (NLOS) signal components. It results in less accurate time synchronization and propagation time measurement, which poses a problem on IPS that relies on signal measurement principles like TOA, TDOA [9], and AOA [10]. Moreover, the RSS is also unstable, owing to the superimposition of multipath signals of varying phases. Meanwhile, the magnetic signal has a very limited discernibility in addition to the requirement of proper calibration of the magnetometer in the smartphone.

Since the indoor environment is much complex and less characterized than the outdoor environment, it is not easy to model the indoor radio signal propagation. The indoor signal propagation model is usually based on a propagation path and the known obstacles where minor indoor changes can render the signal propagation model invalid. The localization methods like trilateration [11] and weighted centroid (WC) localization [12] rely on the signal propagation model to estimate the distance from the RSS. Moreover, these methods require precise calibration of path loss exponent for every indoor environment. Fingerprinting localization is the most widely employed IPS that rely on radio fingerprint to produce localization result. However, this method’s training phase is labor-intensive and time-consuming, and the time complexity of the execution phase grows with the size of the localization area. Moreover, the instability of the RSS in the indoor environment enforces frequent update of the radio map database.

The signal-free localization method relies on mobile sensors such as accelerometers, gyroscope, magnetometer, and barometer and can track the users by continuously estimating their displacement from a known starting point. The necessity of a known starting point and integrated sensor readings to measure a position resulting in an unacceptable accumulated error are dead reckoning-based IPS problems.

As multiple approaches of techniques and technologies have put forward while realizing an IPS, there is no fixed set of rules that guide designing an IPS. In recent years, an initiative has been put forward to set a common benchmark for IPS. For example, the EvAAL framework [13,14] and the Microsoft competition [15] offer researchers a real and challenging test site with independent evaluation. Francesco P. et al. have elaborated on the benchmarks for IPS standardization in [16]. Although IPS are yet to achieve standardization, some research works, such as [5,17,18] that are based on radio frequency (RF), have shown surprising localization accuracy (decimeter level accuracy). In [5], an approach is presented that can compute subnanosecond time-of-flight employing commodity Wi-Fi cards. Here, packets are transmitted on multiple Wi-Fi bands and stitched their information together, mimicking a wideband radio. Reference [17] put forward an approach to jointly estimate the AOA and time-of-flight by combining channel state information (CSI) values across subcarriers and antennas. Similarly, [18] presents the fingerprinting localization based on 60 GHz impulse radio to reduce the effects of NLOS propagation. This work exploits 60 GHz technology’s ability to provide accurate temporal and spatial information for TOA estimation.

A practical IPS should bear the properties like easy implementation, acceptable (meter level) localization accuracy, feasible system cost, scalable/robust system, and minimum computational complexity. However, the localization solutions at hand are more focused only on acquiring better positioning accuracy. In this paper, we discuss the strength and weaknesses of various state-of-art approaches compared with performance metrics of practical wireless technology-based IPS. Therefore, the objective of this survey is to provide a comprehensive outline of available systems and solutions of smartphone-based IPS that uses RF (particularly Wi-Fi and BLE) as its signal source, so that readers may be educated in this rapidly growing area.

This survey paper is organized as follows. Section 2 summarizes some published survey literature on RF-based IPS. In Section 3, a brief description of some RF-based localization technologies and some localization solutions are presented. Signal measurement principles and the performance matrices are elaborated in Section 4 and Section 5, respectively. Section 6 presents the challenges of practical IPS, and Section 7 presents positioning algorithms and a survey of some available solutions. A conclusion is drawn in Section 8 with discussion of future research trends.

## 2. Typical Survey Papers on RF-Based IPS

The available IPS put forward in the past decade are designed for various applications utilizing diverse techniques and technologies. The variations in localization methods, signal sources, and target applications have resulted in a systematical investigation of IPS. However, the indoor localization problem is still waiting for a satisfactory and reliable solution. Meanwhile, the literature on different IPS approaches is summarized in many survey papers based on different research topics [19,20,21,22,23,24,25,26,27,28,29,30]. This section outlines the typical survey papers on IPS to differentiate our work from the existing literature.

Pavel et al. presented a comprehensive study on smartphone-based IPS [19]. The main focus of their discussion is RF and magnetic field-based fingerprinting along with map aided navigation and inertial sensors. They have elaborated on the fingerprinting algorithm in prospective to wireless local area networks (WLAN); however, the significant issues of fingerprinting, such as offline workload and computational complexity, are not discussed. Moreover, this work lacks the evaluation of the IPS based on comparison criteria. The next review on device-based IPS is presented in [22,25], where they deal with seamless outdoors-indoors localization solutions for smartphones and indoor localization for various devices, respectively. As a topic of discussion of [25] is seamless localization, it briefly mentions the approaches of indoor/outdoor localization and their integration and lacks a clear focus on IPS. Similarly, [22] reviews IPS that are realized employing a smartphone and tag devices compatible with various technologies. This work highlights the use of various technologies such as Wi-Fi, BLE, UWB, ultrasound, etc. in IPS and presents their performance comparison. Nevertheless, the localization methods for IPS and their issues are not addressed in the paper.

Another focus of survey papers on IPS is based on the classification of localization systems [21,26,28]. A review of IPS’ multiple techniques and technologies was carried out in [26], which also focuses on the internet of things (IoT). The authors list out the localization system’s applications and tabulate some of the existing IPS to show their advantages and disadvantages. They review the wide variety of techniques and technologies for IPS; however, the presented challenges do not wholly reflect the practical limits and challenges of a pragmatic IPS. Hui Liu et al. have presented a survey of wireless indoor positioning techniques and systems to summarize the positioning algorithms and the existing systems and solutions [28]. This work illustrates an IPS’s performance metrics, which can be a basis for judging any IPS. Besides, a graphical outline of wireless-based positioning systems depicting localization accuracy (resolution), use areas (scale), and wireless technologies is presented. However, this work does not address the challenges and future trends of IPS. A short review of IPSs and the employed algorithms are presented in [21]. Here, a comparison of a few IPSs based on parameters like used technology, accuracy, and robustness are tabulated.

Wi-Fi fingerprinting-based IPS has been neatly reviewed in [29,30]. In [29], Sunning et al. elaborate fingerprinting localization based on spatial and temporal signal patterns wherein they further discuss the various offline workload reduction algorithms for fingerprinting localization. In addition, they summarize the collaborative localization model of IPS that employ distance and proximity-based approaches. Moreover, they review the motion assisted Wi-Fi localization, energy efficiency for smartphones, and list out some future directions. Meanwhile, [30] further elaborates on Wi-Fi-based fingerprinting by formulating the localization problem and detailing the conventional localization approaches. Unlike [29,30] explains the available data clustering modules in IPS for computational complexity reduction. Although both the works are impressive, none of them address other promising technologies (e.g., BLE, etc.) and techniques (e.g., neural network-based fingerprinting, etc.) for fingerprinting localization.

Some other survey papers on IPS are [23,24,27]. The review study in [24] focuses on IPS for emergency responders. Here, the IPS that were designed for emergency responders is categorized as a radio signal, IMU, and hybrid-based approach wherein various localization solutions are discussed. Hakan and Shuang present a survey on IPS that rely on RF, infrared, and ultrasonic technologies [27]. They highlight the benefits of RFID positioning systems. As RSS has been used one way or the other in most of the RF-based IPS, [23] presents a survey paper on IPS that employs CSI wherein the benefits of CSI over RSS are highlighted.

In our survey work, we focus on practical IPSs that rely on RF (Wi-Fi or BLE or both) and smartphones for realizing the real-time localization system. We elaborate on the localization algorithms targeted for such practical IPSs, highlighting the fingerprinting localization due to its promising accuracy. We review the challenges that practically limit an RF-based IPS to achieve all the performance metrics. Moreover, we discuss and tabulate the existing localization solutions that intend to solve a reliable IPS’s single or multiple challenges. Furthermore, we highlight the future research direction in RF-based IPS.

## 3. Wireless Technologies for IPS

**Figure 1:**
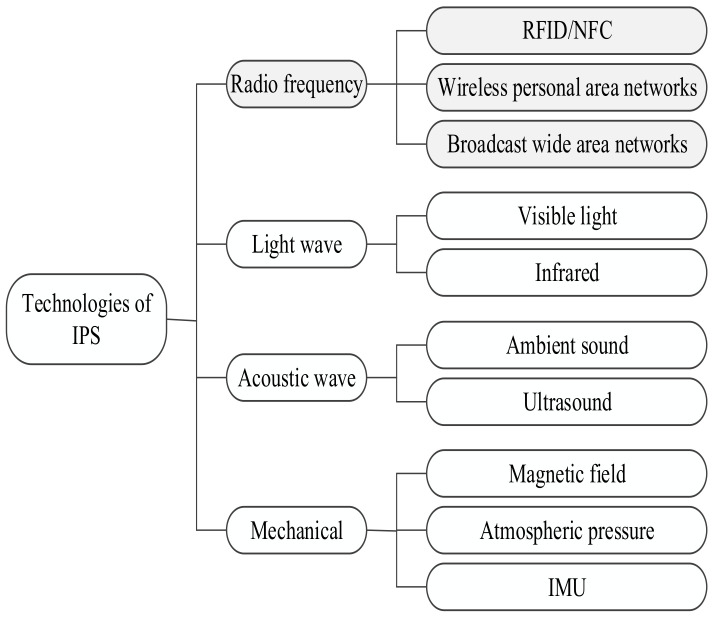
Categorization of major technologies used in indoor positioning system (IPS) development.

Many representative technologies have been employed to develop IPS. The technologies can be categorized into RF, lightwave, acoustic wave, and mechanical, as shown in Figure 1.

RF technologies such as Wi-Fi and BLE are the most used technologies in IPS development. Similarly, the magnetic field, inertial measurement unit (IMU), and atmospheric pressure can be employed for IPS development. Apart from them, lightwave such as visible light and infrared and acoustic waves such as ambient sound and ultrasound are also utilized in indoor localization. Since this survey aims to study IPS approaches based on wireless technologies, an overview of the localization solutions based on some selected RF technologies is emphasized. As Wi-Fi and BLE are the dominating technologies for IPS development, we present their relative comparison in terms of IPS in Table 1.

The RF technology can be further categorized into broadcast wide area network (WAN), wireless personal area network (WPAN), and RFID/NFC. The cellular network, TV/FM radio signals, and GPS repeaters belong to the WAN family, whereas Wi-Fi, BLE, WSN, and UWB belong to the WPAN family.

### 3.1. RFID/NFC

The radio frequency identification (RFID) systems rely on two main components, namely RFID tag and RFID reader to fulfill their objective. The RFID reader wirelessly acquires the electronically stored information of RFID tags. The reader contains a transceiver to transmit RF signals and read the data emitted from the tags. The tags can be categorized as passive and active. The passive tags get energy from incoming radio signals, whereas a battery powers active tags. RFID systems operate in four frequency bands: Low frequency (125 kHz), high frequency (13.56 MHz), ultra-high frequency (433, 868–915 MHz), and microwave frequency (2.45 GHz, 5.8 GHz). The property of RFID to detect and recognize the nearby tag enabled it to be used for IPS.

Some of the RFID localization systems using passive tags are [31,32,33,34] where the tags are deployed on the floor at a fixed distance forming a grid and estimate localization results by detecting multiple tags. The work in [35,36] are based on active tags where RSS is used to estimate the location of the user. RFID has also been combined with other technologies for IPS. For example, [37] combines it with the ultrasonic sensor, [36,38] combine with image sensors to detect the location of objects.

Near Field Communication (NFC) is the short-range (5 cm or less) wireless communication technology. It is mainly a specialized branch within the family of RFID technologies (high-frequency band of RFID). Localization can be realized with NFC by deploying several tags at places of interest, where a location is estimated simply by touching the tag with the NFC equipped device [39,40].

### 3.2. UWB

UWB uses very low energy for short-range and high-bandwidth communications over a large portion of the radio spectrum. In general, an emitted radio wave is considered UWB if its bandwidth exceeds 500 MHz or 20% of the carrier frequency. The properties of UWB, such as very less power consumption, effective penetration through dense materials, and less sensitive to the multipath effect owing to a very short duration of UWB pulses make the UWB suitable for IPS development. As of this writing, Apple (iPhone 11 and 12) and Samsung (Galaxy Note 20 Ultra) have launched their new smartphones that have UWB chip on it.

IPS based on UWB can estimate location accurately owing to the possibility of precise time measurements of the propagation time of UWB pulses. Yanjia et al. proposed a robust method to mitigate the path overlapping effects that induce TOA and AOA based positioning inaccuracy [41]. Their method is based on the spectral observation of beamforming and yields the least-squares estimation of joint TOA and AOA with a low computational cost. As the performance of UWB based IPS deteriorates in the NLOS channel, [42] proposed a method to identify NLOS by measuring signal strengths in the first path and multipath. RSS based UWB IPS system has also been put forward to have an accuracy between 0.1 to 0.2 m [43].

### 3.3. Wireless Sensor Networks

Wireless sensor networks (WSN) are the group of spatially dispersed and dedicated sensor nodes for monitoring and recording the environment’s physical conditions and organizing the collected data at a central location [44,45]. The nodes of WSN are equipped with a processor, storage, a power supply, a transceiver, and one or many sensors, with an actuator. WSN operates at an unlicensed band of 2.4 GHz, and it can use several off-the-shelf wireless technologies like Bluetooth, UWB, and ZigBee where most applications use IEEE 802.15.4 and ZigBee [46]. Some sensor nodes in WSN, called anchor nodes, are aware of their position information. Therefore, the localization problem in WSN-based IPS is to determine the location of other nodes based on location information obtained from the anchor nodes. IPS using WSN normally consists of distance or angle estimation between nodes or their combination to produce localization results.

### 3.4. Wi-Fi

Wi-Fi is a technology for radio wireless local area networking (WLAN) of devices based on 802.11 IEEE network standard, operating in the 2.4 and 5 GHz ISM radio bands. The devices that can use Wi-Fi include PCs, smartphones/tablets, smart TVs, video game consoles, digital audio players, cars, and printers. Wi-Fi is the most popular means of communicating data wirelessly and is increasingly deployed everywhere, including home and public indoor environments. Wi-Fi-based IPS are being intensively studied owing to the widespread deployment of Wi-Fi hot spots.

The localization methods like fingerprinting [47,48] and trilateration [49] can be realized using Wi-Fi. Similarly, signal measurement principles like RSS [50], CSI [51], TOA [52], and their hybrid combination [53] can be used to provide Wi-Fi-based localization service. Although existing Wi-Fi APs can be employed to design a Wi-Fi-based IPS, those Wi-Fi networks are deployed for wireless communication, and localization is not their primary purpose. In other words, the Wi-Fi APs are not dedicated to localization; hence, an enhanced and efficient localization algorithm is required for practical localization results.

Note that Wi-Fi can be used only on the Android platform, and iOS does not provide a public API [54,55] at present. Furthermore, some restrictions regarding permissions and allowed frequency of Wi-Fi scans have been introduced from Android 8.0 (API level 26). Such restrictions have been further tightened in Android 9 (API level 28) and Android 10 (API level 29). For example, in Android 10, a successful call to WifiManager.startScan() requires ACCESS_FINE_LOCATION, ACCESS_COARSE_LOCATION or ACCESS_FINE_LOCATION, and CHANGE_WIFI_STATE permissions [56].

Moreover, a Wi-Fi module usually requires 3–4 s to process the startscan command from the positioning app to acquire a new scan result [57]. In practice, repeated RSS data are acquired until new scan results are produced to perform sampling per second in Wi-Fi AP. In such a case, subsequent filtering must be performed on the obtained Wi-Fi data to remove the erroneous statistical analysis.

### 3.5. BLE

BLE was released as Bluetooth version 4.0 in June 2010. The BLE is designed for devices that do not require large amounts of data transfer and is intended for short-range wireless transmission with low power consumption and cost [46,58]. It is reported that the power draw of the smartphone is lower for BLE than for Wi-Fi [59]. Similar to Wi-Fi, BLE operates at an ISM band of 2.4 GHz. The frequency band is divided into 40 channels spaced at 2 MHz apart, among which the three channels (37, 38, and 39) are used for broadcasting advertisement [60]. It is noteworthy that the three advertising channels are strategically placed to avoid interference with coexisting technologies such as IEEE 802.11 and ZigBee [61]. Moreover, the signal fluctuation in BLE is a consequence of the random use of the advertisement channels [62].

Similar to Wi-Fi, the localization methods like proximity [63], trilateration [64], and fingerprinting [65] can be realized using BLE. The tag device can estimate the RSS from a nearby BLE beacon by intercepting the advertisement packets transmitted by the beacons. The advertisement interval can range from 100 to 2000 ms. The typical advertisement interval of BLE beacons used in IPS is 300 ms by considering the normal walking speed (1.3 m/s). Moreover, the scan interval also can be set in the positioning application. A typical value of the scan interval is 1000 ms (1 s) to produce positioning results every second.

In contrast to Wi-Fi, the BLE beacon is generally compatible with Android and iOS platforms. However, the compatibility may differ depending on different beacon packages, e.g., iBeacon and Eddystone. Apple develops the iBeacon profile (natively supported in iOS), and its signal contains three main pieces of information, namely, UUID, Major, and Minor [66]. Whereas, Eddystone format is developed by Google (with Android users in mind) as an open-source protocol for BLE beacons. The Eddystone broadcasters advertise fields (referred to as frame types), namely, Eddystone-UID (unique static ID), Eddystone-URL (includes a compresses URL), and Eddystone-TLM (telemetry data) [67]. When a beacon region is detected by an iOS application (that monitors iBeacon’s signal), some action (e.g., a push alert to the home screen) is triggered, which can be helpful in real-time scenarios (e.g., stores in a mall). However, iOS does not have such background operating system support for Eddystone triggering [68]. In recent times, cross-platform app SDK like FlutterBlue [69], and Universal Bluetooth Beacon Library [70] have come into the picture that support Android and iOS platforms.

**Table 1:** Comparison of Wi-Fi and Bluetooth Low Energy (BLE) technology in terms of IPS.

Parameters	Wi-Fi	BLE
Deployment cost	Low	High
AP reliability	Not-dedicated to IPS	Dedicated to IPS
Hardware efficiency	Requires ≥ 3 s to scan new RSS data [57]	RSS sample acquired every second
AP differentiating parameters	SSID, BSSID (MAC)	UUID, MAC [65]
Transmission range	High (∼50 m)	Low (∼30 m)
Power consumption [tag]	High [71]	Low [71]
Power source [AP]	Plugged into mains	Powered by coin shaped battery
Channel availability	Three independent channels at most (2.4 GHz band) [57]	Three advertisement channels [72]
Proximity detection	Normally final location estimation is available	Immediate, Near, and Far proximity available [73]
Implementation platform	Only on Android devices [54,55]	iOS and Android devices

### 3.6. Cellular Networks

The cellular network refers to a long-range wireless network distributed over the cells where each cell is served by at least one fixedly located transceiver known as a base station. The cellular network can be classified by the technical standards that have been evolved from 1G (analog) to the latest 5G (digital). The cellular networks-based IPS benefit from cellular signals such as wide coverage, existing infrastructure, multiple frequency bands, and supported by a large number of mobile communication devices.

As the technical standard of cellular networks has evolved with generations, the localization estimation accuracy has also been increased accordingly. For example, the cell-ID localization in 2G helped to improve the accuracy of hundreds to tens of meters [74]. Similarly, localization based on timing via synchronization signals in 3G and reference signals dedicated to localization in 4G has helped increase localization accuracy. Furthermore, it is expected to attain an accuracy of localization estimation in the range of centimeters using 5G-based devices where the 5G networks are expected to use precise localization estimation through all layers of the communication protocol stack [75]. In particular, 5G cellular technology is expected to have a large signal bandwidth (mm-wave) and beamforming capabilities, making the localization more robust and efficient [76].

## 4. Signal Measurement Principles

### 4.1. RSS

The RSS is a measurement of the power present in a received radio signal. The RSS value is measured in decibel-milliwatt (dBm) and has a typical negative value ranging from nearly 0 dBm (excellent signal) to less than −100 dBm (poor signal). As the distance between the transmitter and receiver increases, the RSS gets attenuated due to many factors including the antenna of transmitting and receiving devices, the number of walls and floors, the number of people and furniture, etc. Note that the RSS does not decrease linearly as the distance increases [77].

RSS modeling is usually done by the combined effects of large-scale fading and small-scale fading [78]. The large-scale fading component depicts the signal attenuation as the signal travels over a distance and is absorbed by objects such as walls and floors along the way to the smartphone. This fading component predicts the mean of the RSS and usually has a log-normal distribution [79]. Similarly, the small-scale fading describes the fluctuation of signal due to multipath fading. For the NLOS component, the small-scale fading is modeled with a Rayleigh distribution, whereas, for the LOS component, it is modeled by Rician distribution. In IPS, the fluctuating RSS are filtered using many approaches such as Gaussian filter [80], moving average filter [12,81], and exponential averaging [82].

Owing to walls and other objects between the transmitter and the receiver, NLOS signals in an indoor environment are common, which can significantly degrade the localization accuracy. The RSS, while used in parameter estimation (e.g., path loss exponent) methods, is converted to distance employing a path-loss model. In a practical indoor environment, the localization scenario is so complex (includes both LOS and NLOS signal propagation) that it is difficult to establish an accurate model to work. On the other hand, the fingerprinting localization that stores the RSS as a radio map is less sensitive to the NLOS conditions [83]. Reference [84] classified the methods for reducing the NLOS error as direct and indirect methods. Here, the direct method refers to directly processing the measurement results reducing the NLOS propagation error employing Kalman filtering, particle filtering, etc. The indirect method refers to the fingerprinting localization.

RSS value can be acquired without any extra hardware using off-the-shelf smartphones. Moreover, RSS does not require any time synchronization between the transmitter and the receiver. Most importantly, RSS values can be implied to realize any indoor positioning methods, where it can be converted to distance for lateral approaches and stored in a database for scene analysis. Hence, RSS has been a prime choice of signal measurement principle in IPS.

### 4.2. TOA

**Figure 2:**
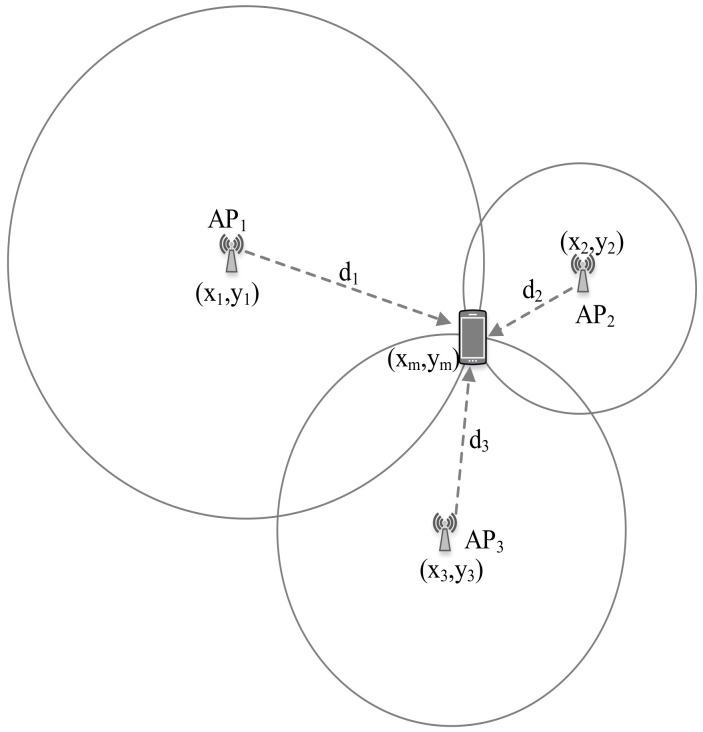
Localization based on time of arrival (TOA) measurement.

The TOA is the travel time or time of flight of a radio signal from a transmitter to a receiver. As the signal travels with a known velocity, the distance can be directly calculated from the TOA. Figure 2 illustrates a TOA measurement-based localization system.

Let *c* be the speed of light, then the distance between *i*th AP and the tag device can be estimated by the following relation [85]:

(1) $$d_{i}=\left(t_{i}-t_{0}\right)\times c,$$

where $t_{0}$ and $t_{i}$ are the time instant of signal transmission and signal reception respectively, and $c=3\times 10^{8}$ m/s. The TOA technique requires precise time synchronization for transmitters and receivers. The estimated distance can be utilized for the trilateration algorithm to estimate user location. TOA has been used with various wireless technologies like UWB [86] and Wi-Fi [87].

### 4.3. TDOA

**Figure 3:**
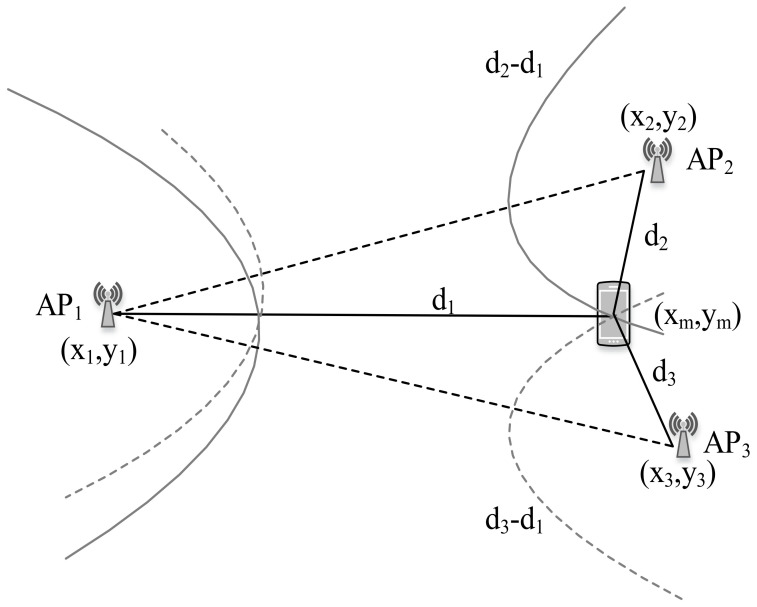
Localization based on time difference of arrival (TDOA) measurement.

For TDOA measurement, the difference in arrival time from multiple APs is employed. In TDOA based localization, the distance difference between the tag device and APs is calculated based on time difference measurements as shown in Figure 3.

Here, the difference of distance to APs and to the AP where the signal first arrives is [88]:

(2) $$d_{ij}=\left(t_{i}-t_{j}\right)c=\sqrt{{\left(x_{i}-x_{m}\right)}^{2}+{\left(y_{i}-y_{m}\right)}^{2}}-\sqrt{{\left(x_{j}-x_{m}\right)}^{2}+{\left(y_{j}-y_{m}\right)}^{2}},$$

where $t_{i}$ and $t_{j}$ are the time instant of signal reception from AP *i* and *j*, respectively. Geometrically, with a given TDOA measurement, the tag device must lie on a hyperboloid with a constant range difference between the two APs. Apart from TOA, TDOA needs time synchronization of APs only. Besides, the timing measurements at TOA and TDOA can be achieved down to a fraction of chip duration assuming a LOS condition; however, NLOS can cause information loss during the time measurement. To incorporate the NLOS condition, [89] suggests modeling the error as a mixture of LOS and NLOS models for a robust algorithm. Furthermore, [90,91] put forward a three-dimensional least-square positioning technique and NLOS error estimation approach for positioning in the NLOS environment, respectively.

### 4.4. AOA

**Figure 4:**
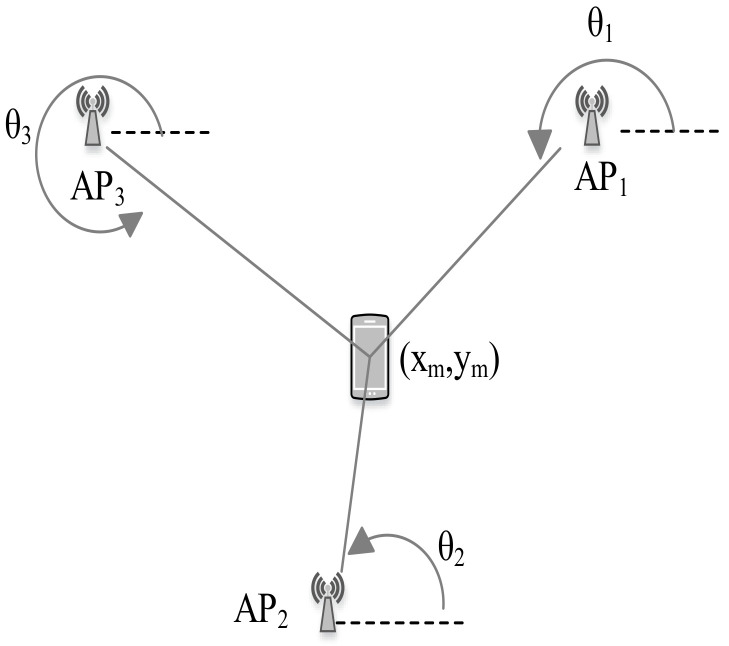
Localization based on angle of arrival (AOA) measurement.

The AOA information is extracted employing the directionally sensitive antennas [89]. The AOA measurement determines the direction of propagation of a radio wave incident on an antenna array. It can be done by measuring the TDOA at individual elements of the antenna array [92]. AOA-based localization system estimates the location of the tag device as the intersection point of pairs of hypothetical signal paths particular angles as shown in Figure 4.

At the 2D plane, the AOA approach requires only two APs to determine the location of a tag device [93]. In AOA-based IPS, time synchronization between the APs and the tag device is not required. However, it may require relatively complex hardware to obtain angle measurement [94]. For instance, Ubicarse [95] leverages antenna arrays and computes AOA of signals from the APs to estimate the device orientation.

### 4.5. CSI and RTT

The CSI is an emerging approach that tries to replace RSS information in IPS [23,96,97,98]. The CSI explains how a signal propagates from a transmitter to the receiver. In other words, it describes the information that represents a combined effect of scattering, fading, and power decay with the distance. It is reported that CSI achieves higher robustness compared to Wi-Fi RSS information [99]. Moreover, the SpotFi combines CSI values across subcarriers and antennas to jointly estimate the AOA and time-of-flight for decimeter level localization [17]. However, current smartphones are not compatible with CSI data collection, making it impossible to implement CSI-based positioning solutions on the present-day smartphone.

The RTT stands for Round Trip Time that can measure the distance without requiring time synchronization between the communicating nodes. The time spent by a specific frame is measured while traveling from a transmitter to a receiver and back again to the transmitter. Wi-Fi RTT was introduced in Android 9 (API level 28), which is specified by IEEE 802.11 mc standard and built on a new packet type known as fine timing measurement (FTM) frame [100]. Here, the Wi-Fi RTT API provides Wi-Fi location functionality to measure the distance to nearby RTT-capable Wi-Fi APs and peer Wi-Fi Aware devices [101].

**Figure 5:**
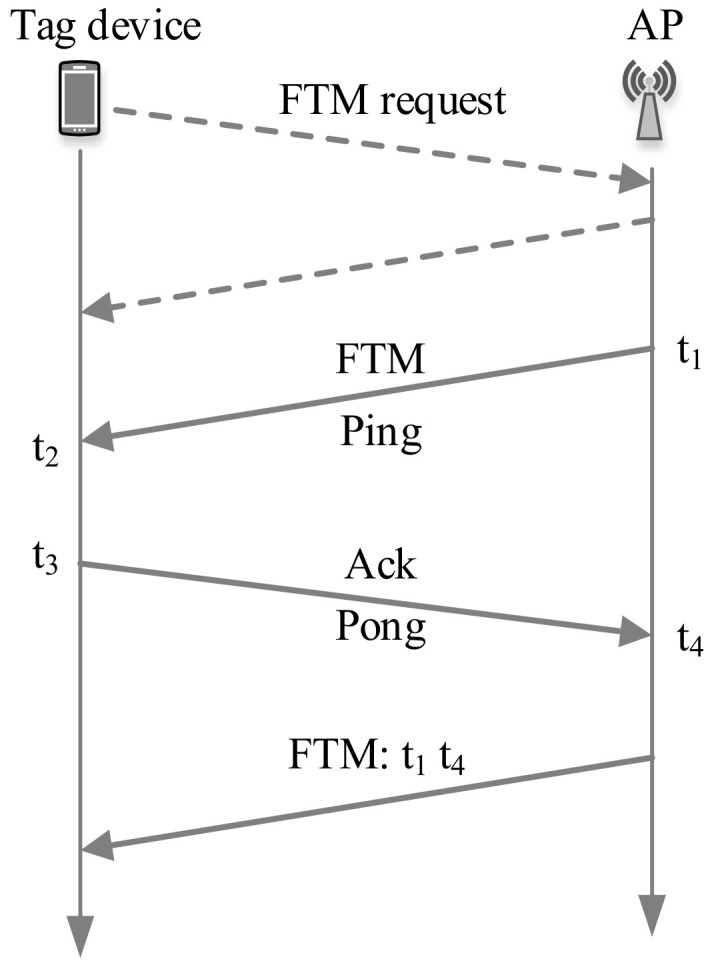
The fine timing measurement (FTM) protocol.

The FTM protocol is shown in Figure 5, where a tag device initiates the FTM process by sending an FTM request to an AP. The AP (that supports FTM protocol) responds to the FTM request either to agree or to disagree with the ranging process. If the AP agrees, it starts to send an FTM message, waits for its acknowledgment (ACK), and transmits the FTM result afterward. The transmitting timestamp of the FTM message and the reception timestamp of its ACK is utilized to infer the propagation delay between the tag device and the AP. Here, the AP can send multiple FTM messages for averaging the estimated distances [102]. Table 2 differentiates FTM- and UWB-based approaches.

From Figure 5, RTT is calculated for an FTM message as [104,105]

(3) $$RTT=\left(t_{4}-t_{1}\right)-\left(t_{3}-t_{2}\right)$$

The distance ($d_{RTT}$) between the transmitter and receiver can be estimated by multiplying the RTT with the speed of light (*c*) as follows

(4) $$d_{RTT}=\frac{RTT}{2}\times c$$

Multiple trilaterations can be employed with the estimated distance to localize a tag device. The main challenge on RTT is the NLOS that increases uncertainty in the time measurement [106]. It can be minimized by using LOS/NLOS identification approaches [107,108].

## 5. The Performance Metrics

Since there are different technologies and methods to realize an IPS, the most important performance metric is the localization accuracy. In addition to localization accuracy, other performance indicators of an IPS are complexity, scalability/robustness, and cost.

### 5.1. Accuracy and Precision

The localization accuracy can be defined as a difference between an estimated location and the tag device’s actual location. Similarly, the precision indicates the degree to which repeated location estimates produce identical results under unchanged conditions.

Usually, the mean squared error (MSE) is used as the accuracy indicator. Indoor environments are often complex due to different obstacles and environmental changes, resulting in signal fluctuation. In the meantime, high localization accuracy is often expected for adequate location-based service. The localization accuracy of the IPS depends on the used technology and techniques.

The precision yields information like how convergent the localization result can be over many trials or how consistently the system works. The cumulative distribution function (CDF) is used as a precision indicator. The CDF represents the distance error distribution between the estimated location and the tag device’s actual location. The MSE, as well as CDF, should be exploited while comparing two or more localization algorithms.

### 5.2. Complexity

For an IPS, its complexity can be categorized in terms of hardware and software. The adopted technology and the signal measurement principle for the IPS account for hardware complexity. For example, most present-day smartphones supported technologies like Wi-Fi, BLE, and the geomagnetic field. However, standard mobile devices do not support technologies like UWB and ultrasound, and the IPS using such technologies should use a dedicated system that requires proprietary equipment. Moreover, geomagnetic based IPS can produce localization results without deploying any hardware; however, Wi-Fi and BLE need to be deployed.

As for the chosen signal measurement principle, obtaining RSS from Wi-Fi and BLE are relatively easy with standard mobile phones since such devices typically need to scan RSS for their routine functioning. However, it is not easy to obtain accurate time and angle measurements that increase the IPS complexity.

The complexity depends on the computation load represented by the calculations required to perform localization regarding the software. In a server-based IPS, the localization algorithm’s execution is carried out on a centralized server where the positioning could be calculated quickly due to its powerful processing capability and abundant power supply. Here, the computational complexity mainly depends on the indoor localization area [109]. However, if the positioning algorithm is executed in the tag device, it may increase the complexity. Moreover, the complexity concerned with the software also depends on the technique used for IPS development. For example, fingerprinting localization has larger complexity, and it grows as the localization environment increases. Here, the complexity can be minimized using clustering.

### 5.3. Scalability and Robustness

Scalability in IPS refers to the localization system’s ability to perform well even when any change in the area of interest for localization and/or on signal source occurs. The changes can be an extension of the localization area and/or an extension of signal coverage. If an IPS need not be taken down in such a scenario, it is considered excellent scalability. For example, when any IPS is constructed, it provides services in a limited area of interest, and an increase in the localization area might be needed in some future time. In some cases, the transmitting power and signal-broadcasting rate can be increased for good signal coverage. In such situations, the positioning techniques like proximity, WCL, and trilateration are easy to expand by merely adding the identical signal sources and updating the system with the location coordinate information of the added hardware. However, the fingerprinting based system needs an offline site survey for every change in the localization area or signal source. When the localization area is expanded, the extended area’s radio map needs to be freshly constructed. Moreover, when the transmitting power at the APs or signal broadcasting rate (e.g., advertisement packet broadcasting interval in BLE) is changed, a new site survey for the whole localization area is required. Hence, fingerprinting localization has relatively low scalability.

Robustness is also an essential factor in IPS that allows the system to function normally without human intervention when the localization environment changes. For example, some signal sources could be out of service occasionally, or testbed layout changes could cause some signal to no longer support LOS propagation. In this scenario, the IPS has to provide localization services with incomplete or noisy information (RSS fluctuation). The robustness can be gained by introducing redundant information into a localization estimation. For example, rather than using the only three APs for trilateration, an IPS can include many supplementary APs to make the system more robust. Moreover, fingerprinting based IPS can adopt a larger set of RSS samples to increase robustness.

### 5.4. Cost

The IPS cost factor represents the infrastructure cost and the time and effort for system installation and maintenance procedures. Particularly, the cost depends on factors like the size of the localization area, the required accuracy, the used technology, power consumption, etc. The IPS system, such as PDR, geomagnetic-based, and barometer-based, can be realized with only the smartphone and do not need any additional infrastructure. Some signal sources like Wi-Fi and BLE require APs deployment; however, Wi-Fi is already deployed for other purposes. Hence, if the IPS requires no additional infrastructure or is based on existing infrastructure, the cost can be substantially saved.

Moreover, if an IPS is robust and scalable, saving in time and labor is possible, which reduces the system’s cost. In addition, an increase in localization space requires the deployment of more APs in localization techniques like proximity. Power consumption at both the AP and the tag device is also a critical cost issue. For example, BLE consumes less power than Wi-Fi, where BLE operates with a coin-shaped battery, but Wi-Fi needs to be plugged into mains. Furthermore, when the localization operation is carried out on the server-side, the tag device’s power consumption can be reduced. Lastly, the location update rates, signal broadcasting rate, and the desired system accuracy can also affect power consumption.

## 6. The Problems of Practical IPS

### 6.1. Complex Indoor Environment and Unstable RSS

**Figure 6:**
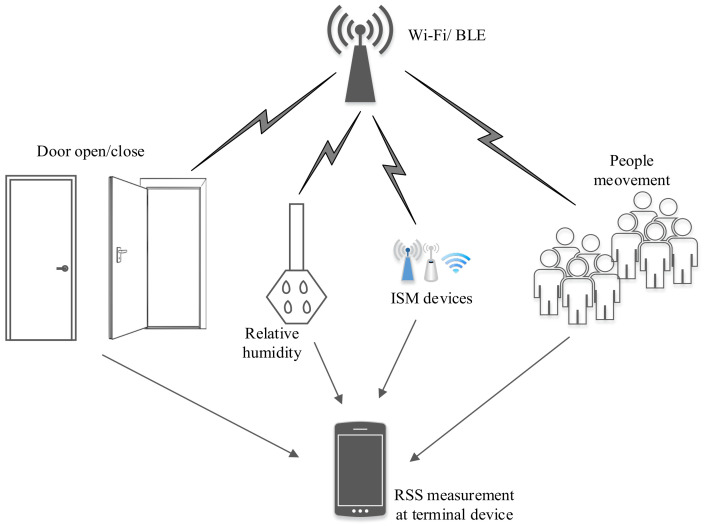
Graphical representation of interference at indoor environment.

**Figure 7:**
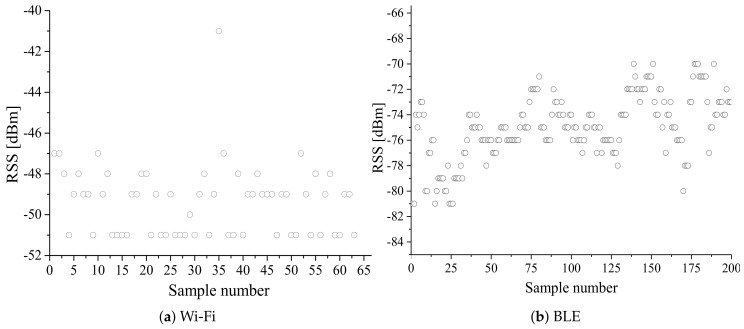
Variation of Wi-Fi and BLE RSS at a fixed indoor environment.

The indoor area consists of multiple floors, walls, furniture, and human beings, which results in a complex radio environment. Hence, RSS exhibits high variability in space and time, even in a fixed indoor environment owing to various noise factors, interferences, and attenuation. A probable radio environment at an indoor location is displayed in Figure 6. This instability of RSS results in increased localization estimation error. Therefore, irrespective of the localization technique, RSS filtration/smoothing is required to minimize the localization estimation error. Figure 7 shows the fluctuation of Wi-Fi and BLE RSS in a fixed indoor environment (without any change in the radio environment).

From Figure 7, it is seen that RSS fluctuates a lot even at a fixed point in the indoor environment. To minimize this problem, a low-pass smoothing filter can be employed. Some of the representative smoothing filters are moving average filter [12,81], Kalman filter [110,111,112], Gaussian filter [72,113], and exponential averaging [114].

### 6.2. Terminal Device Heterogeneity and Battery Efficiency

Most positioning techniques require either calibrated environmental parameters or the radio map construction to provide localization service. Such parameters/radio-map, when calibrated/constructed by a terminal device and localized by another kind of terminal device, may result in an adverse effect on position estimation owing to the different gains of receivers and antennas [115,116,117]. For example, the calibrated signal strength at a unit distance for the log-distance path loss model by two different terminal devices may not yield the same value. Similarly, the stored signal strength for any RP on a testbed acquired by a terminal device may differ by certain decibels when measured by a different terminal device at the same measurement place. Therefore, it is required to address the issue of terminal device heterogeneity while designing a practical IPS.

The available indoor localization solutions intend to provide high accuracy service with the terminal device’s consumption of high battery energy. The existing IPSs require active monitoring of the wireless channels to listen to specific beacon message or advertisement signals periodically. On top of this, some IPS integrate different technologies for better positioning results. For example, Reference [118] employs the magnetic field, PDR, and QR code and the BLE signals to enhance the localization estimation. While it is practical performance-wise, it is not ideal in terms of battery efficiency. Note that localization service is not the primary task of any terminal device; hence, the battery drainage can lead to consumer dissatisfaction. Thus, it is worthwhile to focus on energy consumption simultaneously to the localization accuracy.

### 6.3. Learning Methodology of Radio Signals in Scene Analysis

The fingerprinting localization technique is predominantly realized in IPS applications owing to its high reliability. However, the learning methodology of the radio signals in fingerprinting localization is costly in terms of time-consumption and workload. In other words, a data collector has to visit every hypothetical grid or the RP that is typically one meter apart to acquire the offline training dataset. Generally, the training dataset at an RP consists of an average of RSS samples (typically 35 samples) from each AP along with the RP’s coordinate. Moreover, the radio map has to be updated repeatedly owing to the RSS fluctuation and possible change in the radio environment. This issue on fingerprinting-based localization intensifies practical limits and challenges in realizing a reliable and scalable IPS to meet the required accuracy of practical IPS. Many research works have endeavored to reduce the offline workload of fingerprinting localization [119,120]. Some of the approaches are the use of a self-guided robot, simultaneous localization and mapping (SLAM) [121,122], machine learning [123,124], and crowdsourcing [125,126]. In addition, the signal propagation model has been employed for generating a fingerprinting database to reduce the offline workload [127,128,129]. However, an efficient solution for the data collection problem is yet to discover.

### 6.4. Computational Time and System Cost

Apart from good positioning accuracy, fast position estimation is also favored for a practical IPS. Typically, a new position estimation at each second is demanded. Hence, computational time should also be considered while improvising the positioning method for better positioning results [130]. For example, the positioning result of probability-based fingerprinting is better than deterministic-based fingerprinting (Wk-NN); however, the computational complexity of the probabilistic approach of fingerprinting is higher than the deterministic approach [30]. Furthermore, as two or more technologies are integrated for better localization solution, the computational complexity increases.

Not every novel and efficient localization algorithm may be commercially successful owing to the system cost. Considering the Wi-Fi and BLE, Wi-Fi APs are already deployed in every building for communication purposes, whereas BLE needs to be deployed that increases the system cost. Furthermore, positioning methods requiring lengthy data learning and frequent data updating procedures raise the system cost. The increased system cost can make the concerned parties reluctant to adopt IPS for better service.

## 7. Positioning Algorithms and Survey of Available Solutions

This section presents some positioning algorithms, focusing on fingerprinting localization and summarizing various state-of-art approaches intended to solve IPS problems. A comparison of available systems and solutions against the performance metrics is presented in Table 3.

### 7.1. Proximity-Based

**Figure 8:**
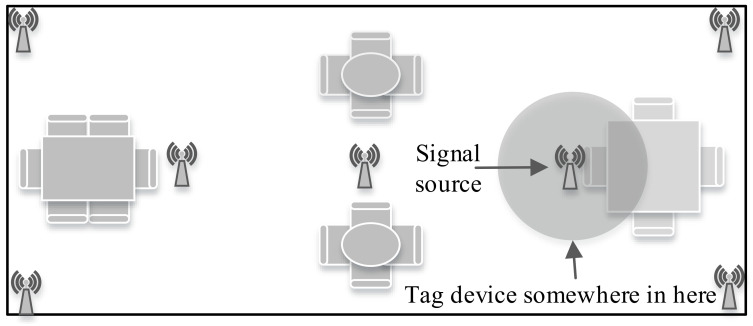
Graphical representation of proximity-based IPS.

The word proximity is defined as nearness in space, time, or relationship. As the definition suggests, proximity in IPS provides symbolic location information if an object is present within an AP’s vicinity where the received signal strength determines the vicinity. A proximity-based IPS is illustrated in Figure 8.

In proximity-based IPS, when a tag device detects an AP, the tag’s position is associated with the AP’s location. In this scenario, when the tag device detects more than one APs in its vicinity, the tag device’s location can be referred to the AP’s real location having the strongest signal. The proximity-based IPS is the simplest among all the algorithms and is very easy to implement. However, for better localization accuracy or high resolution, a dense deployment of APs is mandatory. Generally, the IPS using wireless technologies like WSN, BLE [131,132], RFID [133], and NFC [39] is employed for proximity-based IPS development. Reference [134] uses pedestrian dead reckoning (PDR) and BLE beacons to estimate the user location. This work employs the proximity information of the beacons for correction of the estimated position of PDR.

### 7.2. Lateral/Angular

The lateral technique estimates the position of a tag device by measuring the distances from multiple APs. The distance can be obtained from signal measuring principles like RSS, TOA, and TDOA. Similarly, the angulation technique estimates the tag device’s location by computing angles relative to multiple APs using AOA. WC localization and trilateration are the lateral methods, whereas triangulation is the angulation method.

#### 7.2.1. WC Localization

In proximity-based IPS, when the tag device detects multiple APs in its vicinity, the location can be estimated as a centroid of the real location of the detected APs. Furthermore, a certain weight can be assigned to each detected AP based on their signal strength to estimate a weighted centroid. The simplest WC localization equation is defined by the following set of equations [12]:

(5) $$\begin{array}{c} x_{w}=\frac{\sum_{j=1}^{u}x_{j}\times w_{j}}{\sum_{j=1}^{u}w_{j}}\\ y_{w}=\frac{\sum_{j=1}^{u}y_{j}\times w_{j}}{\sum_{j=1}^{u}w_{j}}\\ w_{j}=\frac{1}{d_{j}^{g}}, \end{array}$$

where ($x_{w},y_{w}$) is the estimated WC, ($x_{j},y_{j}$) is the previously known AP coordinate, $d_{j}$ is the estimated distance between the tag device and *j*th AP, *g* is the degree of weight, and *u* is the total number of APs considered for WC localization.

**Figure 9:**
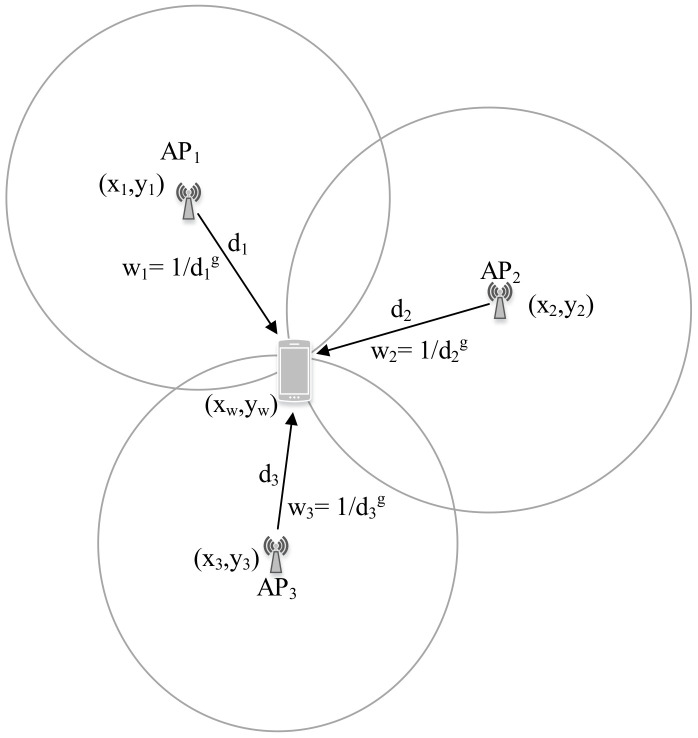
Procedure for estimating weighted centroid (WC) localization at a tag device.

Figure 9 illustrates the WC localization procedure.

The WC localization has the following characteristics [72]:

1. The estimated location is confined inside the APs’ real location only.
2. The estimated location is dragged towards the nearest AP owing to its largest weight.

The degree of weight (*g*) can be adjusted as per the distance between the deployed APs. A large value of *g* drives the WC location very close to the real location of the AP with the strongest signal, whereas a very low value (close to zero) yields a geometrical centroid among the *u* APs.

#### 7.2.2. Trilateration

Trilateration is based on measured distances between a tag device and several APs with their known real location coordinates. Given the distance to an AP, it is known that the tag device must be along the circumference of a circle centered at the AP and radius equal to the tag-AP distance. For a 2D localization, at least three noncollinear APs are needed, whereas, for 3D localization, at least four noncoplanar APs are required to perform trilateration operation.

Let us consider *B* APs with their real location coordinate $x_{i}$ = ($x_{i},y_{i}$ ) (i=1,2,....B) and unknown location of the tag device be x = (x,y). The distances between the tag device and the APs is $d_{i}$ (i=1,2,…,B). The relationship between APs/tag positions and their distances in 2D can be written as [135]:

(6) $$\left[\begin{array}{c} {\left(x_{1}-x\right)}^{2}+{\left(y_{1}-y\right)}^{2}\\ {\left(x_{2}-x\right)}^{2}+{\left(y_{2}-y\right)}^{2}\\ \vdots \\ {\left(x_{B}-x\right)}^{2}+{\left(y_{B}-y\right)}^{2} \end{array}\right]=\left[\begin{array}{c} d_{1}^{2}\\ d_{2}^{2}\\ \vdots \\ d_{B}^{2} \end{array}\right]$$

Equation (6) can be represented as *A*x = *b* where *A* and *b* are defined as:

(7) $$A=\left[\begin{array}{cc} 2(x_{B}-x_{1}) & 2(y_{B}-y_{1})\\ 2(x_{B}-x_{2}) & 2(y_{B}-y_{2})\\ \vdots & \vdots \\ 2(x_{B}-x_{B-1}) & 2(y_{B}-y_{B-1}) \end{array}\right]$$

(8) $$b=\left[\begin{array}{c} d_{1}^{2}-d_{B}^{2}-x_{1}^{2}-y_{1}^{2}+x_{B}^{2}+y_{B}^{2}\\ d_{2}^{2}-d_{B}^{2}-x_{2}^{2}-y_{2}^{2}+x_{B}^{2}+y_{B}^{2}\\ \vdots \\ d_{B-1}^{2}-d_{B}^{2}-x_{B-1}^{2}-y_{B-1}^{2}+x_{B}^{2}+y_{B}^{2} \end{array}\right]$$

The location of the tag device can be estimated based on the least squares system using $x={\left(A^{T}A\right)}^{-1}A^{T}b$ [136].

#### 7.2.3. Triangulation

In contrast to trilateration, triangulation uses angle measurements in addition to distance measurements to estimate the position of the tag device. Two angles and one length are required for a 2D localization. Particularly, triangulation utilizes the geometric properties of triangles to estimate the tag location.

**Figure 10:**
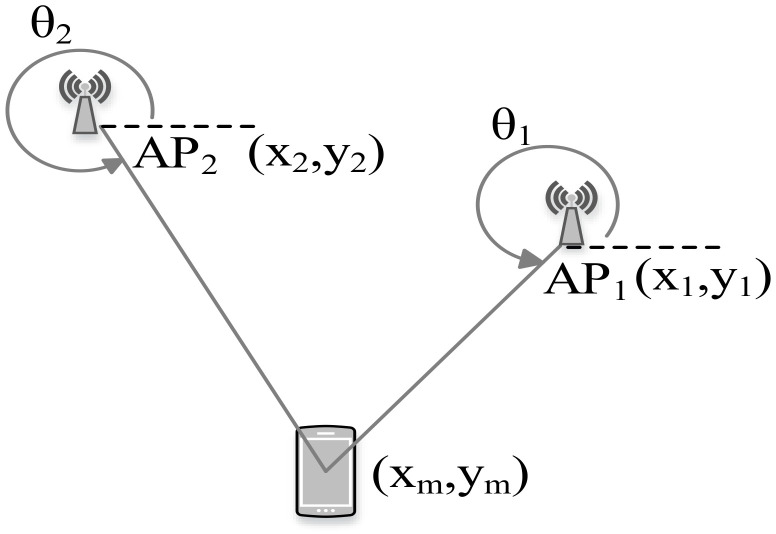
Triangulation localization using two known APs and AOA.

Given the known length between the APs (known location coordinates of the APs) and after estimating the AOA as shown in Figure 10, the location of the tag device can be estimated as follows [137]:

(9) $$\begin{array}{c} x_{m}=\frac{y_{2}-y_{1}+x_{1}\tan\theta_{1}-x_{2}\tan\theta_{2}}{\tan\theta_{1}-\tan\theta_{2}}\\ y_{m}=y_{2}-\frac{\left(x_{2}-x_{1}\right)\tan\theta_{1}-\left(y_{2}-y_{1}\right)}{\tan\theta_{1}-\tan\theta_{2}}\tan\theta_{2}, \end{array}$$

where $\theta_{1}$ and $\theta_{2}$ are the estimated angle of incident at two APs with their location coordinates $\left(x_{1},y_{1}\right)$ and $\left(x_{2},y_{2}\right)$, respectively.

### 7.3. Fingerprinting

**Figure 11:**
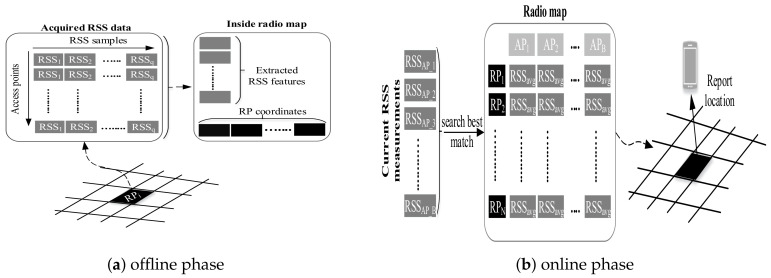
The working procedure of typical fingerprinting localization.

Fingerprinting is also called scene analysis, where signal strength at reference points (RPs) is measured and stored in the database along with the location of the coordinate of the RPs. For localization, new signal strength is measured and compared with the saved ones to estimate a location. Hence, a fingerprinting localization has two phases of operations as illustrated by Figure 11. In the offline phase, the area of interest is divided into nonoverlapping hypothetical grids. The typical grid size is 1 m. The data collector goes from one grid to another to collect RSS from hearable APs. In the online phase, the freshly acquired RSS is compared to the stored one to estimate the user’s position.

Fingerprinting is the most widely used indoor localization method due to its good localization accuracy and nonrequirement of LOS measurements of APs. The technologies like Wi-Fi, BLE, and geomagnetic field can be used to realize the fingerprinting localization. Although fingerprinting localization has good localization accuracy, it comes with a time-consuming and labor-intensive offline phase. In particular, conventional fingerprinting localization can be categorized as deterministic and probabilistic. The former approach implements fingerprinting data comparison algorithms to find the estimated position, whereas the latter approach yields localization information by estimating a probability distribution over the RPs. In addition, neural networks have also been utilized resembling the scene analysis to produce localization estimation.

#### 7.3.1. Deterministic Fingerprinting Localization

In deterministic fingerprinting localization, the observed RSS is compared against the stored one in the database, and then the coordinate with the closest match is considered the estimated tag device’s location. This approach is the basic fingerprinting that is commonly termed as the nearest neighbor (NN) fingerprinting localization [138]. To improvise the localization result, *k*-nearest neighbor (KNN) can be employed where *k* nearest RPs are selected based on their closest match for an enhanced result [130,139]. Furthermore, individual weights can be assigned to the selected *k* RPs to have a weighted *k*-nearest neighbor (Wk-NN) fingerprinting [72]. Let the positioning distance ($D_{j}$) between the stored RSS at *j*th RP and the online observed RSS be given by [140]:

(10) $$\begin{array}{c} D_{j}=\sum_{i=1}^{B}\sqrt{{\left(RSS_{i_{online}}-RSS_{i_{offline}}\right)}^{2}}\;j=1,2,.....,N, \end{array}$$

where *i* is the number of APs ranging from 1 to *B*. The RPs are arranged with ascending order of $D_{j}$ and the first *k* RPs with their known positions $J_{z}\left[x_{z},y_{z}\right]$ are selected to estimate the final location ($T_{Wk-NN}$) using the following relation [141]:

(11) $$\begin{array}{c} T_{Wk-NN}=\frac{\sum_{z=1}^{k}J_{z}\times W_{z}}{\sum_{z=1}^{k}W_{z}},\;\mathrm{where}\;W_{z}=\frac{1}{D_{z}} \end{array}$$

#### 7.3.2. Probabilistic Fingerprinting Localization

The probabilistic approach of fingerprinting yields localization information by estimating a probability distribution over the RPs. Here, a matching probability is calculated between the online-observed RSS readings and the prestored fingerprinting data in the radio map database.

Since the statistical distribution of RSS at a particular RP can be thought of as Gaussian probability distribution, the RSS values should obey the normal distribution *N* ($\mu ,\sigma^{2}$) where μ and $\sigma^{2}$ are mean and variance of RSS data [142], respectively. Now, the likelihood function ($L(\mu ,\sigma^{2})$) is given by the following relation [143]:

(12) $$\begin{array}{c} L\left(\mu ,\sigma^{2}\right)=\prod_{i=1}^{q}\frac{1}{\sqrt{2\pi }\sigma }\exp^{-\frac{{\left(RSS_{i}-\mu \right)}^{2}}{2\sigma^{2}}}={\left(2\pi \sigma^{2}\right)}^{\frac{-q}{2}}\exp^{\left(-\frac{1}{2\sigma^{2}}\sum_{i=1}^{q}{\left(RSS_{i}-\mu \right)}^{2}\right)} \end{array}$$

We can obtain the logarithmic equation of (12) as follows:

(13) $$\begin{array}{c} \log\left[L\left(\mu ,\sigma^{2}\right)\right]=-\frac{q}{2}\log\left(2q\right)-\frac{q}{2}\log\left(\sigma^{2}\right)-\frac{q}{2\sigma^{2}}\sum_{i=1}^{q}{\left(RSS_{i}-\mu \right)}^{2} \end{array}$$

Hence, the likelihood equations can be written as:

(14) $$\begin{array}{c} \frac{\partial \log[L\left(\mu ,\sigma^{2}\right)]}{\partial \mu }=\frac{1}{\sigma^{2}}\sum_{i=1}^{q}{\left(RSS_{i}-\mu \right)}^{2}=0\\ \frac{\partial \log[L\left(\mu ,\sigma^{2}\right)]}{\partial \sigma^{2}}=-\frac{q}{2\sigma^{2}}+\frac{1}{2\sigma^{4}}\sum_{i=1}^{q}{\left(RSS_{i}-\mu \right)}^{2}=0 \end{array}$$

From (14), we get,

(15) $$\begin{array}{c} \mu *=\bar{RSS}=\frac{1}{q}\sum_{i=1}^{q}RSS_{i} \end{array}$$

(16) $$\begin{array}{c} \sigma^{*2}=\frac{1}{q}\sum_{i=1}^{q}{\left(RSS_{i}-\mu \right)}^{2} \end{array}$$

The unique solution ($\mu^{*},\sigma^{*2}$) of the likelihood equations should also be a local maximum point. In other words, when $\left|\mu \right|$→∞ or $\sigma^{2}$→∞ or $\sigma^{2}$→0, the non-negative function $L(\mu ,\sigma^{2})\to 0$. Hence, the maximum likelihood equation of μ and $\sigma^{2}$ will be (15) and (16), respectively.

Hence, with this idea, the average of RSS reading and its corresponding variance from each AP is calculated for the construction of a radio map in the offline phase of the probability-based fingerprinting localization. Later, in the online phase, after acquiring new RSS readings ($RSS_{i},i=1,2,3,\ldots ,B$) at an unknown location from *B* APs, we can estimate the probability of the RP (x,y) with respect to *i*th AP ($P_{i}\left(x,y\right)$) as follows:

(17) $$\begin{array}{c} P_{i}\left(x,y\right)=\frac{1}{\sqrt{2\pi }\sigma_{i}}\exp^{\frac{-{\left(RSS_{i}-\mu \right)}^{2}}{2\sigma_{i}^{2}}}, \end{array}$$

where $\mu_{i}$ and $\sigma_{i}^{2}$ are the stored average RSS and its corresponding variance from the *i*th AP, respectively. This way, the probability of each RP can be established where the tag’s location could be located at the RP with maximum probability.

#### 7.3.3. Neural Networks-Based Fingerprinting Localization

**Figure 12:**
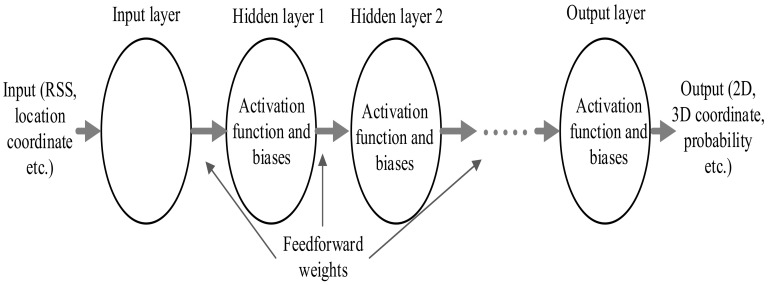
A typical architecture of multilayer perception (MLP).

The indoor localization problem has been attempted to solve using neural networks too. The radio map is used as inputs and the targets for training the neural network during the offline state to obtain the appropriate weights. In the online stage, the input signal strength is multiplied with a trained weight matrix at different layers to yield either 2D/3D location or probabilities depending on the activation function at the output layer. In general, an artificial neural network (ANN) with one hidden layer (shallow) or multiple hidden layers (deep) can be employed in neural networks based localization. The basic architecture of multilayer perception (MLP) is given in Figure 12.

In [144], an indoor localization solution based on deep neural networks (DNN) is presented. This work employs a four-layered DNN-based probabilistic estimator and RSS preprocessing, where the raw RSS is normalized to the range between 0 to 1. For offline DNN training, the preprocessed RSS readings of the radio map database are used. Online positioning consists of two layers: coarse and fine positioning. At coarse positioning, trained DNN is used to yield probabilities of all the RPs, and the Hidden Markov model is used to refine the coarse positioning estimate. ANN-based fingerprinting is used in [145] to estimate the location coordinate. This work uses clustering where a separate ANN is trained for each cluster or region.

#### 7.3.4. Survey of Available Fingerprinting Localization Solutions

- **Fingerprinting localization using Wi-Fi and BLE signals**: Pavel et al. present an IPS research work based on Wk-NN positioning method using BLE beacons [146]. The *k*-nearest fingerprints are found in a radio map database by employing the Euclidean distance between the observed RSS and the database’s referred one. This work further compares the localization methods based on Wi-Fi and a combination of BLE and Wi-Fi. They recommend that the combination of wireless technologies help to increase the localization accuracy. Next work based on BLE beacons using fingerprinting technique is reported in [80] where a Gaussian filter is used to preprocess the received RSS. This work proposes a distance-weighted filter based on the triangle theorem of trilateral relations to filter out the wrong distance value caused by an abnormal RSS.
  The traditional Wk-NN fingerprinting has also been realized with Wi-Fi signals. Reference [29] elaborates recent advances on Wi-Fi fingerprinting localization. They overview advanced localization techniques and efficient system development utilizing Wi-Fi technology in their survey work. An improvisation over the conventional Wk-NN fingerprinting using Wi-Fi signals is put forward in [147,148]. The former approach uses average RSS and standard deviation of Wi-Fi signals at the RPs from the APs to construct a fingerprint radio map. Both the average RSS and the standard deviation are processed to estimate a Euclidean distance in the online phase. With the Euclidean distance, *k* RPs are selected to estimate a coarse location. Furthermore, a joint probability for each RP is calculated, based on which the *k* RPs are selected to estimate another coarse location. Later, both the coarse localization estimations are fused, employing the shortest Euclidean distance and the largest joint probability to yield a final localization estimation. Meanwhile, the later approach proposes to use Manhattan distance instead of Euclidean distance to compare the closeness of acquired Wi-Fi signal strength with the stored database.
  Some of the examples of probability-based fingerprinting localization using Wi-Fi as a signal source are illustrated in [123,142,149,150]. Similarly, the literature that employ BLE for probability-based fingerprinting are presented in [59,151].
- **Machine learning-based methods**: The machine learning algorithm extracts valuable information from the raw data and represents it as a model or hypothesis, which can be used for other unseen data to infer things. Although Gaussian process regression (GPR) is widely used in geostatistics as a Bayesian kringing, it has drawn a lot of attention in the machine learning community in recent decades. The GPR can be defined as a supervised learning task, which can predict the RSSs at arbitrary coordinates based on acquired training data. The prediction of RSS across the testbed with little training data helps to reduce the human workload significantly. Reference [123] presents a GPR-based fingerprinting IPS using indoor Wi-Fi APs. This work uses a few data points to train the Gaussian process (GP), where the firefly algorithm is used to estimate the GP’s hyperparameters. Moreover, it also shows that the probabilistic-based localization performs better than deterministic-based localization using the predicted radio map. Liu et al. proposed a GPR-plus method with Bluetooth transmitters using a naïve Bayes algorithm [152]. They compare their method with [123] and claim that their method is computationally cheaper. Another example of GPR-based fingerprinting is put forward in [149]. This work estimated the hyperparameters by using the subspace trust-region method and shows that location estimation with a radio map built using GPR is better than that of Horus fingerprinting method [153]. The GPR-based IPS in [151] utilizes BLE beacons for localization where the Hlhyperparameters are optimized employing limited memory BFGS-B [154]. Here, the predicted RSS data is further preprocessed for RSS clustering, where the final localization result is obtained with the minimized offline workload and reduced online computational complexity.
  In [155], the use of a support vector machine (SVM) is proposed to estimate the Wi-Fi signal strength at non-sight-surveyed locations on the testbed. This system creates an RSS reference surface for each AP using discrete train data with SVM. During the testing phase, the sampled online RSS from each AP is searched on the corresponding surfaces. Here, the coordinate that is found in the higher number of such surfaces is estimated as the tag device’s location.
- **Crowdsourcing techniques**: Although machine learning approaches like GPR are intended to solve the offline workload problem, they still require a little training data that are manually acquired from the localization area. Hence, recent literature on solving the offline workload problem of fingerprinting localization is more focused on the crowdsourcing [156,157]. Here, the main concept is to crowdsource the RSS data from freely moving users across the testbed. It is straightforward to understand that unlabeled RSS data are easy to acquire from various users. However, the main concern is to find a plausible way to label the crowdsourced RSS data with the ground-truth location.
  In [158], a smartphone-based crowdsourcing approach is proposed that employs an accelerometer as a pedometer. Here, multidimensional scaling (MDS) is used to create a map that displays the relative positions of several objects employing only a table of distance values among them. The walking distance between two RPs is estimated using the accelerometer to form a distance matrix. The MDS utilizes the distance matrix as its input to map all the RPs into a *d*-dimensional Euclidean space forming a "stress-free" floor plan. Meanwhile, a next distance matrix is also formed utilizing the walking distance between two fingerprint positions, where again the MDS maps all fingerprints to a *d*-dimensional Euclidean space to form a fingerprint space. Finally, the stress-free floor plan and the fingerprint space are mapped to form a radio map database. X. Tong et al. suggested a FineLoc system for indoor radio map construction employing BLE beacons and PDR as the source of reference information [159]. The FineLoc system generated the tag’s trace and then determines the map for the trace. For online positioning, this system merges the tag’s trace into the existing floorplan.
  Similarly, [160] has put forward a trajectory learning method utilizing crowdsourcing measurements to support the absence of a map. Here, the k-nearest neighbor is used to perform a classification model with linear discriminant analysis (LDA) and principal component analysis (PCA) for floor detection. The combination of LDA and PCA employs the acquired training data to make a classification model. Moreover, [161] uses a commercial software called Trusted Positioning Navigator (T-PN) for crowdsourcing based IPS. This method forms a crowdsourced fingerprinting database employing the RSS values and position information from the T-PN software.
- **Clustering-based approaches**: The conventional fingerprinting is also termed as flat fingerprinting and can be converted to two-step fingerprinting using clustering or segmentation.The two-step fingerprinting is realized with a coarse localization step and fine localization step, as the name suggests. Clustering reduces the searching space of RPs in the online phase of fingerprinting, which eventually reduces the system’s computational cost. Moreover, it also helps to reduce the localization estimation error by removing the outliers.
  Clustering on IPS can be realized using either hardware (Wi-Fi or BLE) or the RSS clustering. An example of a clustering module using hardware is Horus [153]. Here, the clustering module is employed where any cluster is a set of RPs sharing a common set of Wi-Fi APs. This approach estimates the tag’s position based on the largest posterior probability by Bayesian interference [162]. Similarly, [163] uses BLE beacon proximity to reduce the searching space in the online phase. Here, BLE’s proximity provides coarse localization, and for fine localization, a selected set of RPs is used with Wi-Fi fingerprint datasets.
  The performance of indoor fingerprinting positioning can be improved with RSS clustering [30]. An RSS clustering method chooses a set of cluster centers to reduce the sum of squared distances between the RSS value and their corresponding centers. For example, a K-means clustering [164] begins by choosing both the number of output clusters and the corresponding set of initial cluster heads, where the clustering algorithm iteratively refines the output clusters to decrease the sum of squared distances [165]. Hence, K-means clustering has a requirement of an arbitrary selection of initial cluster centers. On the other hand, affinity propagation clustering (APC) starts by assigning each point (RP in this study) the same chance to become a cluster center where all the points are joined in the large space [166]. Reference [167] uses APC for clustering the testbed using Wi-Fi RSS data. Here, the cluster-head is determined on the coarse localization, and Wk-NN is used for fine localization. In addition to APC and K-means, other clustering methods in IPS include fuzzy c-means and hierarchical clustering strategy (HCS) [168,169,170,171]. APC has been a widely used clustering technique in IPS owing to its initialization-independent and better cluster head selection characteristics. Many kinds of literature on IPS have employed APC for RSS clustering where their fine localization is either probabilistic-based or deterministic-based [150,167,172,173].

**Table 3:** Wireless technology (Wi-Fi and BLE)-based localization system and solution.

[-0.5+]System	[-0.5+]Tech.	[-0.5+]Signal	[-0.5+]Positioning Algorithm	Performance Metrics					
				Accuracy	Precision	Complexity	Scalability/ Space Dimension	Robustness	Cost
Horus [174]	Wi-Fi	RSS	Probabilistic method	2 m	90% (2.1 m)	Moderate	Good/2D	Good	Low
RADAR [175]	Wi-Fi	RSS	deterministic method	3–5 m	50% (2.5 m), 90% (5.9 m)	Moderate	Good/2D,3D	Good	Low
Robot assistive [176]	Wi-Fi	RSS	Bayesian approach	1.5 m	Over 50% (1.5 m)	Medium	Good/2D	Good	Medium
Ekahau [177]	Wi-Fi	RSS	Probabilistic method	1 m	50% (2 m)	Moderate	Good/2D	Good	Low
IPS in [178]	Wi-Fi, IMU	RSS	Fingerprinting+PDR	2.4 m	88% (3 m)	High	Good/2D	Weak	Low
IPS in [123]	Wi-Fi	RSS	Probabilistic method	3 m	90% (9 m)	Moderate	Excellent/2D	Good	Low
IPS in [149]	Wi-Fi	RSS	Probabilistic method	2.3 m	N/A	Moderate	Excellent/2D	Good	Low
IPS in [179]	Wi-Fi, IMU	RSS	Fingerprinting+PDR	2.2 m	90% (1 m)	High	Good/2D	Weak	Low
IPS in [124]	Wi-Fi	RSS	Support vector regression	0.68 m	90% (1.4 m)	Moderate	Excellent/2D	Good	Low
IPS in [150]	Wi-Fi	RSS	Probabilistic method	2.23 m	80% (3 m)	Moderate	Good/2D	Weak	Low
IPS in [172]	Wi-Fi	RSS	Probabilistic method	1.94 m	94% (3 m)	Moderate	Good/2D	Weak	Low
IPS in [180]	BLE, IMU	RSS	Proximity+PDR	0.28 m	N/A	High	Excellent/2D	Weak	Medium
IPS in [72]	BLE	RSS	WCL+Fingerprinting	1.6 m	90% (2 m)	Moderate	Good/2D	Good	Medium
IPS in [134]	BLE, IMU	RSS	Proximity+PDR	2.26 m	N/A	High	Good/2D	Weak	Medium
IPS in [59]	BLE	RSS	Probabilistic method	N/A	95% (2.6 m)	Moderate	Good/2D	Good	Medium
IPS in [151]	BLE	RSS	Probabilistic method	2.25 m	76% (3 m)	Moderate	Excellent/2D	Good	Medium
IPS in [181]	BLE, IMU	RSS	Fingerprinting+PDR	0.65 m	95% (1.5 m)	High	Good/2D	Weak	Medium
IPS in [114]	BLE	RSS	WCL+Fingerprinting	1.06 m	95% (1.5 m)	Moderate	Good/2D	Good	Medium
IPS in [182]	BLE	RSS	Rank-based fingerprinting	0.78 m	70% (1 m)	Moderate	Good/2D	Good	Medium
IPS in [118]	BLE, Magnetic field, IMU	RSS, Magnetic Flux	Wk-NN+PDR	1.31 m	85% (2 m)	Very High	Good/2D	Weak	Low
FineLoc [159]	BLE IMU	RSS	Crowdsourcing, trace merge, PDR	1.21 m	80% (1.57 m)	High	Excellent/2D	Good	Medium
LiFS [158]	Wi-Fi, IMU	RSS	Crowdsourcing, deterministic method	2 m	90% (4 m)	High	Excellent/2D	Good	Low
IPS in [125]	BLE	RSS	Crowdsourcing, probabilistic method	2.34 m	76% (4 m)	Moderate	Excellent/2D	Good	Medium

## 8. Conclusions and Future Research Trends

We conduct this survey to review the RF-based indoor localization solutions presented in recent literature. As modern smartphones and wireless technologies are evolving, the paper focuses more on practical IPS realized in a smartphone and uses Wi-Fi and BLE as their primary signal source. We described different available wireless technologies and techniques for IPS development. Furthermore, the paper explains the different performance metrics of IPS and their trade-off. As the available localization solutions focus on achieving good localization accuracy/precision, their computational complexity should not be forgotten.

Fingerprinting localization is a promising technique of IPS. However, it is doomed by the requirement of the offline training phase. Although machine-learning and crowdsourcing approaches are put forward to solve the issue of data collection, the IPS still awaits feasible solutions. The RF-based wireless technologies, particularly Wi-Fi and BLE, are widely used for indoor LBS owing to their characteristics like signal penetration, power consumption, localization accuracy, and convenient deployment.

Future research direction on RF-based IPS may lead towards a hybrid system integrating multiple techniques and alternative technologies, efficient learning methodology of radio signals, and deep learning approaches. As 5G technology is rising across the world and UWB chip is available on the latest smartphones, future IPS can exploit and integrate these technologies to develop a better IPS. Future research works can be focused on finding a better solution for easy data acquisition. For example, machine-learning/hardware (Wi-Fi, BLE, and UWB) assistive crowdsourcing approaches are worth considering. Since DNN is used successfully in designing IPS, DNN-based IPS is also a good research topic. Moreover, as there is a lack of standardization (a set of rules that serve as a guide to designing an IPS), there is also no fixed wireless technology widely accepted as a major technology for future IPS. Most of the available systems and solutions are disjoint, where no ubiquitous IPS exists. Hence, the IPS requires a standardization that can narrow down the techniques and technologies, which fulfills the performance metrics of practical IPS.

We believe that the timely and comprehensive overview of the recent works in this survey will further encourage new research efforts into the practical IPS.

## Figures and Tables

**Table 2:** Comparison of fine timing measurement (FTM)- and ultra-wideband (UWB)-based approaches.

Parameters	FTM-Based Approach	UWB-Based Approach
Time transfer	Reference Broadcast Infrastructure Synchronization (RBIS)	Precision Time Protocol (PTP)
Ranging	Fine Timing Measurement (FTM)	Two-Way Ranging (TWR)
Cost	Low	High
Power consumption	High	Low
Distance estimation accuracy	>1 m [103]	5–10 cm
Smartphone compatibility	Wi-Fi RTT introduced in Android 9 (API level 28)	Samsung Galaxy Note 20 Ultra and iPhone 11/12 contain a chip for UWB
